# 铂类联合多西他赛或长春瑞滨一线治疗晚期非小细胞肺癌的*meta*分析

**DOI:** 10.3779/j.issn.1009-3419.2014.04.07

**Published:** 2014-04-20

**Authors:** 太省 刘, 华 吴, 贤勉 庄, 笛 卢, 瑞君 蔡, 武军 王

**Affiliations:** 510515 广州，南方医科大学南方医院胸心血管外科 Department of Thoracic and Cardiovascular Surgery, Nanfang Hospital, Southern Medical University, Guangzhou 510515, China

**Keywords:** 多西他赛, 长春瑞滨, 铂类, 肺肿瘤, *meta*分析, Docetaxel, Vinorelbine, Platinum, Lung neoplasms, *Meta*-analysis

## Abstract

**背景与目的:**

以铂类为基础联合第三代药物的双药化疗方案是治疗晚期非小细胞肺癌（non-small cell lung cancer, NSCLC）的标准一线治疗方案。本研究采用*meta*分析的方法评价多西他赛联合铂类（docetaxel plus platinum, DP）方案对比长春瑞滨联合铂类（vinorelbine plus platinum, VP）方案治疗晚期NSCLC的疗效和安全性。

**方法:**

计算机检索Pubmed、EMBASE、Cochrane Library、中国期刊全文数据库（CNKI）、中国生物医学文献数据库（CBM）、中文科技期刊全文数据（VIP）库及万方数据库关于DP方案与VP方案治疗晚期NSCLC的随机对照试验（randomized controlled trial, RCT）。根据Cochrane Handbook 5.1.0的质量评价标准，用Stata 12.0软件进行统计学分析。

**结果:**

研究共纳入7项RCTs，包括晚期NSCLC患者2, 381例。DP方案的2年生存率（HR=0.887, 95%CI: 0.810-0.972, *P*=0.010）、有效率（RR=1.276, 95%CI: 1.107-1.450, *P*=0.001）和腹泻发生率（RR=3.134, 95%CI: 1.918-5.121, *P* < 0.001）较VP方案高；DP方案减少了贫血的发生率（RR=0.386, 95%CI: 0.311-0.478, *P* < 0.001）；DP方案与VP方案在1年生存率、白细胞减少、中性粒细胞减少、血小板减少、厌食、恶心、呕吐方面的差异无统计学意义。

**结论:**

DP方案虽然增加了腹泻发生率，但却减少了贫血的发生率，同时提高了2年生存率和有效率。相比VP方案，DP方案可能更适合一线治疗晚期NSCLC。

## Abstract (en)

**Background and objective:**

Platinum plus a third-generation agent doublet chemotherapy is the standard regimen and first-line chemotherapy for the treatment of advanced non-small cell lung cancer (NSCLC). The aim of this study is to evaluate the efficacy and safety of docetaxel plus platinum (DP) compared with vinorelbine plus platinum (VP) regimens in patients with advanced NSCLC.

**Methods:**

We searched the PubMed, EMBASE, Cochrane Library, CNKI, CBM, VIP, and WanFang databases for randomized controlled trials (RCTs), in which DP regimen was compared with VP regimen. A quality assessment of qualified RCTs was performed according to Cochrane Handbook 5.1.0, and Stata 12.0 was used to perform *meta*-analysis.

**Results:**

Seven RCTs involving 2, 318 patients were included. *Meta*-analysis results indicated that the DP regimen increased the two-year survival rate (HR=0.887, 95%CI: 0.810-0.972, *P*=0.010), response rate (RR=1.276, 95%CI: 1.107 -1.450, *P*=0.001), and diarrhea rate (RR=3.134, 95%CI: 1.918-5.121, *P* < 0.001) compared with the VP regimen. Anemia rate was also reduced (RR=0.386, 95%CI: 0.311-0.478, *P* < 0.001). No statistical differences were observed between DP and VP regimens in terms of one-year survival rate, leukopenia, neutropenia, thrombocytopenia, anorexia, nausea, and vomiting.

**Conclusion:**

DP regimen reduced the rate of anemia and increased the rate of diarrhea, two-year survival rate, and response rate. Therefore, DP regimen may be a more effective option as a first-line treatment for advanced NSCLC compared with VP regimen.

肺癌是全球癌症和癌症相关死亡的首要原因，每年约120万人死于肺癌^[1]^。非小细胞肺癌（non-small cell lung cancer, NSCLC）约占肺癌的80%，其中20%-30%为局部晚期（Ⅲ期），40%-50%已有远处转移（Ⅳ期）^[2]^。以铂类为基础的双药方案是治疗NSCLC的标准方案^[3]^。近年来，已证明铂类联合第三代化疗药物（长春瑞滨、多西他赛、紫杉醇、吉西他滨等）比传统化疗更有效^[4]^。VP方案是第一种被证实可以提高晚期NSCLC生存率的第三代化疗方案，中位生存时间8.2个月-9.0个月，因而成为治疗晚期NSCLC的一线方案，然而毒性作用较大^[5-7]^。所以仍然有必要寻找既能提高生存率和生活质量并能降低毒性作用的化疗药物。多西他赛是目前欧盟和美国批准唯一一种既可一线也可二线治疗NSCLC的药物。Ⅱ期和Ⅲ期临床试验^[8-10]^显示DP方案一线治疗NSCLC的有效率为30%-67%，中位生存时间为8.4个月-13.9个月，表明DP方案同样可以作为治疗NSCLC的一线方案。然而哪种第三代化疗药物联合铂类最有效、最能延长患者的生存时间且毒性较小一直存在争议^[11]^。

DP方案是否比VP方案具有更好的疗效及安全性，是否能取代VP方案成为治疗晚期NSCLC的一线方案，目前尚无定论。本研究参照Cochrane系统评价的方法，对所有关于DP方案和VP方案一线治疗晚期NSCLC的疗效和副作用进行评价，以期为临床决策提供参考依据。

## 1 资料与方法

### 1.1 纳入与排除标准

#### 1.1.1 研究类型

随机对照试验（randomized controlled trial, RCT），无论是否采用盲法。

#### 1.1.2 研究对象

纳入标准：①经病理组织学或细胞学确诊的Ⅲb期、Ⅳ期的原发性NSCLC患者；②既往未接受过抗肿瘤治疗；③卡氏评分（Karnofsky, KPS）≥60分或者美国东部肿瘤协作组-体力状况（Eastern Cooperative Oncology Group performance status, ECOG PS）评分≤2分；④化疗前心、肝、肾功能及心电图等未见明显异常。排除标准：①信息无法利用及数据无法提取的文献；②利用相同研究人群发表的多篇文献只纳入其中介绍最详细的一篇；③同时伴有其他恶性肿瘤；④伴有严重内科疾病或感染。

#### 1.1.3 干预措施

多西他赛联合铂类（顺铂或卡铂）*vs*（长春瑞滨联合铂类顺铂或卡铂）。

#### 1.1.4 结局指标

主要结局指标：1年生存率、2年生存率、有效率；次要结局指标：毒副作用发生率。疗效根据Therasse等^[12]^提出的RECIST标准（Response Evaluation Criteria in Solid Tumors）分为：完全缓解（complete response, CR）、部分缓解（partial response, PR）、稳定（stable disease, SD）和进展（progressive disease, PD）。有效率（response rate, RR）等于完全缓解率+部分缓解率（CR+PR）。毒副作用按照美国国立癌症研究所公共毒性标准（National Cancer Institute Common Toxicity Criteria, NCI-CTC）。

### 1.2 检索策略

以“（docetaxel OR taxotere）AND（vinorelbine OR navelbine）AND（platinum OR cisplatin OR carboplatin OR oxaliplatin OR nedaplatin OR lobaplatin）AND（non-small cell lung cancer or NSCLC）检索Pubmed（1966-2013.11）、EMBASE（1974-2013.11）和Cochrane Library（2013年第10期）”等外文数据库，以“（多西他赛OR泰索帝）AND（长春瑞滨OR诺维本）AND（铂类OR顺铂OR卡铂OR奥沙利铂OR奈达铂OR洛铂）AND（非小细胞肺癌OR非小细胞肿瘤OR NSCLC）”检索检索中国期刊全文数据库（CNKI, 1994.1-2013.11）、中国生物医学文献数据库（CBM, 1978.1-2013.11）、中文科技期刊全文数据库（VIP, 1989.1-2013.11）及万方数据库（1982.1-2013.11）。RCTs检索策略遵循Cochrane系统评价指导手册5.1.0，所有检索均采用主题词与自由词相结合的方式，并根据具体数据库调整，所有检索策略均通过多次预检索后确定。并用Google Scholar、Medical Martix等在互联网上查找相关文献，对纳入文献的参考文献进行扩大检索，与本领域的专家、通讯作者等联系以获取以上检索未发现的相关信息。

### 1.3 文献筛选和资料提取

两位研究员交叉核对纳入研究的结果，若遇分歧则通过讨论解决并由第三位研究员决定是否纳入。提取的数据主要包括：①一般资料：题目、第一作者姓名、发表年份和文献来源等；②研究特征：研究对象的一般情况、各组病人的基线可比性、干预措施；③结局指标：1年生存率、2年生存率、有效率，治疗引起的并发症包括血液学毒性、消化道毒性、衰弱乏力等。

### 1.4 质量评价

按照Cochrane系统评价指导手册5.1.0评价RCT质量的评价标准^[13]^，质量评价主要涉及以下几个方面：①随机分配方法；②分配方案隐藏；③对研究对象、治疗方案实施者和研究结果测量者采用盲法；④结果数据的完整性；⑤选择性报告研究结果；⑥其它偏倚来源。针对每一项研究结果，对上述6条内容做出“是”（低度偏倚）、“否”（高度偏倚）和“不清楚”（缺乏相关信息或偏倚情况不确定）的判断。

### 1.5 统计分析

对收集的数据采用Stata 12.0进行*meta*分析。生存率资料采用风险比（hazard ratio, HR）为疗效分析统计量；计数资料采用相对危险度（relative risk, RR）为疗效分析统计量；计量资料采用加权均数差（weighted mean difference, WMD）或标准化均数差（standard mean difference, SMD），各效应量均以95%可信区间（confidence interval, CI)表示。各纳入研究结果间的异质性采用χ^2^检验。当各研究间无统计学异质性（*P* > 0.1, *I*^2^ < 50%）时，采用固定效应模型（fixed effect model）对各研究进行*meta*分析；如各研究间存在统计学异质性（*P* < 0.1, *I*^2^ > 50%）时，则分析其异质性来源，对可能导致异质性的因素进行亚组分析，若两项研究组之间存在统计学异质性而无临床异质性或差异无统计学意义时，采用随机效应模型（random effect model）进行分析。

### 1.6 发表偏倚的评价

使用基于*Begg’s*检验为基础的漏斗图（funnel plot）检测数据之间是否存在发表偏倚，并采用*Egger’s*检验进行计算^[14]^。

## 2 结果

### 2.1 文献检索结果

按照检索策略和资料收集方法，共查到相关文献434篇，通过排除重复或不符合纳入标准的文献，可能符合标准的文献有15篇，再经过阅读全文筛选出7项^[15-21]^合格的RCTs。文章的筛选过程见图 1，7项RCTs共纳入2, 381例患者，进入分析的研究两组基线资料均具有可比性，一般情况见表 1。

**1:**
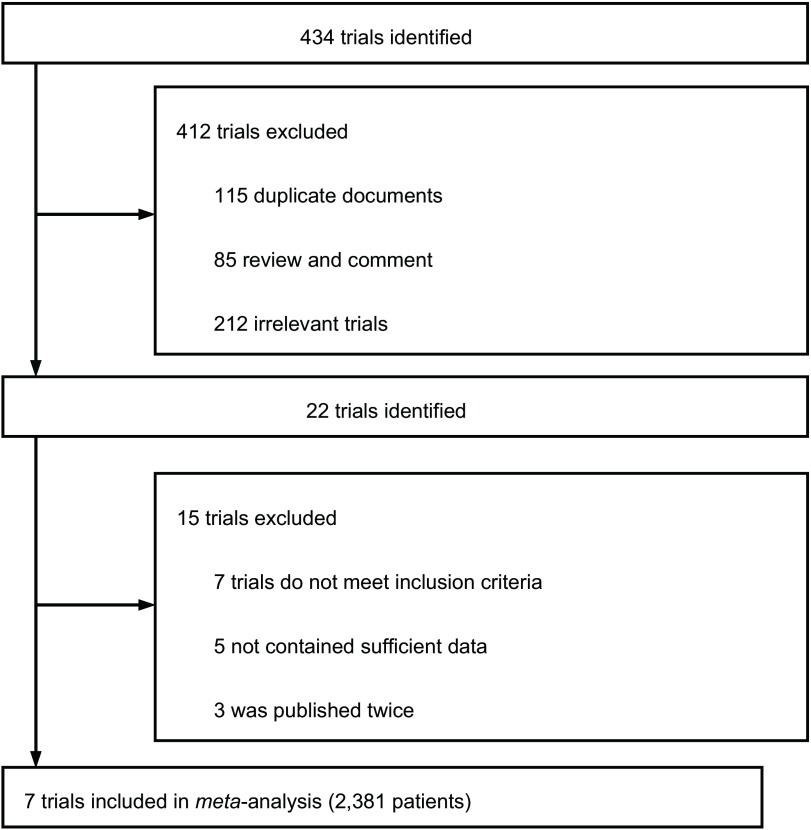
纳入研究流程图 Selection of trials

**1:** 纳入研究的基本特征 The basic characteristics of the studies enrolled

Included study	Sponsor/Country	Follow-up (month)	No. of Patients			Median Age (yr)			Disease stage (Ⅲb/Ⅳ)			Sex (M/F)		Docetaxel (Regimen and dose)	Vinorelbine (Regimen and dose)
			DP	VP		DP	VP		DP	VP		DP	VP		
Fossella 2003^[15]^	American	10	814	404		60	61		267/547	133/271		586/228	302/102	D, 75 mg/m^2^ day 1; C, 75 mg/m^2^ day 1 q 3 wk×6; 292: D, 75 mg/m^2^ day 1; Cb AUC 6 mg/mL.min day 1 q3wk ×6	V, 25 mg/m^2^ days 1, 8, 15, 22; C, 100 mg/m^2^ day 1 q 4 wk ×6
Kubota 2004^[16]^	Japan	12	151	151		63	64		0/151	0/151		97/54	103/48	D, 60 mg/m^2^ day 1; C, 80 mg/m^2^ day 1 q 3 or 4 wk × ≥ 2 cycles	V, 3 mg/m^2^ days 1, 8, 15; C, 80 mg/m^2^ day 1 q 4 wk × ≥ 2 cycles
Douillard 2005^[17]^	France	8.8	115	118		58	57		0/115	0/118		96/19	95/3	D, 75 mg/m^2^ day 1; C, 100 mg/m^2^ day 1 q 3 wk ×6	V, 30 mg/m^2^ days 1, 8; C, 100 mg/m^2^ day 1 q 3 wk ×6
Chen 2007^[18]^	Taiwan	18	46	48		60.2	64.9		9/37	8/40		26/20	35/13	D, 60 mg/m^2^ day 1; C, 60 mg/m^2^ day 1 q 3 wk ×4-6	V, 25 mg/m^2^ days 1, 8; C, 100 mg/m^2^ day 1 q 3 wk ×4-6
Song 2007^[19]^	China	3-25	32	35		61	62		20/12	22/13		27/5	27/8	D, 37.5 mg/m^2^ day 1; C, 80 mg/m^2^ day 1, 2, 3 q 3 wk ×4	V, 25 mg/m^2^ days 1, 8; C, 80 mg/m^2^ day 1, 2, 3 q 3 wk ×4
Tan 2009^[20]^	Singapore	12	191	190		62.1	59.4		29/162	37/153		146/45	139/51	D, 75 mg/m^2^ day 1; C, 75 mg/m^2^ day 1 q 3 wk ×6	V, 25 mg/m^2^ *i.v.* day 1, 60 mg/m^2^ oral day 8; C, 80 mg/m^2^ day 1 q 3 wk ×6
Gebbia 2010^[21]^	Italy	-	42	44		61	62		8/34	9/35		32/10	35/9	D, 75 mg/m^2^ day 1; C, 75 mg/m^2^ day 1 q 3 wk ×6	V, 30 mg/m^2^ days 1, 8; C, 80 mg/m^2^ day 1 q 3 wk ×6
D: docetaxel; P: platinum; V: vinorelbine; C: cisplatin; Cb: carboplatin; AUC: area under curve; M: Male; F: Female.															

### 2.2 纳入研究的质量评价

纳入7项RCTs中，7项研究均提到随机分组，其中2项研究^[15, 21]^随机序号由计算机数字生成器产生，其余均未提及；7项研究均未提及分配隐藏；只有一项研究^[16]^采用双盲，其余均为提及；1项研究^[19]^未报道数据缺失且未进行意向性分析（intention-to-treat analysis, ITT），其余6项研究均有提及（表 2）。

**2:** 纳入研究的质量评价 Quality evaluation of included trials

Included study	Randomized method	Allocation concealment	Blinding	Incompleteness of data	Selective outcome reporting	Other sources of bias
Fossella 2003^[15]^	Yes	Unclear	Unclear	Yes	Unclear	Unclear
Kubota 2004^[16]^	Unclear	Unclear	Double-blind	Yes	Unclear	Unclear
Douillard 2005^[17]^	Unclear	Unclear	Unclear	Yes	Unclear	Unclear
Chen 2007^[18]^	Unclear	Unclear	Unclear	Yes	Unclear	Unclear
Song 2007^[19]^	Unclear	Unclear	Unclear	No	Unclear	Unclear
Tan 2009^[20]^	Yes	Unclear	Unclear	Yes	Unclear	Unclear
Gebbia 2010^[21]^	Unclear	Unclear	Unclear	Yes	Unclear	Unclear

### 2.3 *meta*分析结果

#### 2.3.1 1年生存率（图 2）

**2:**
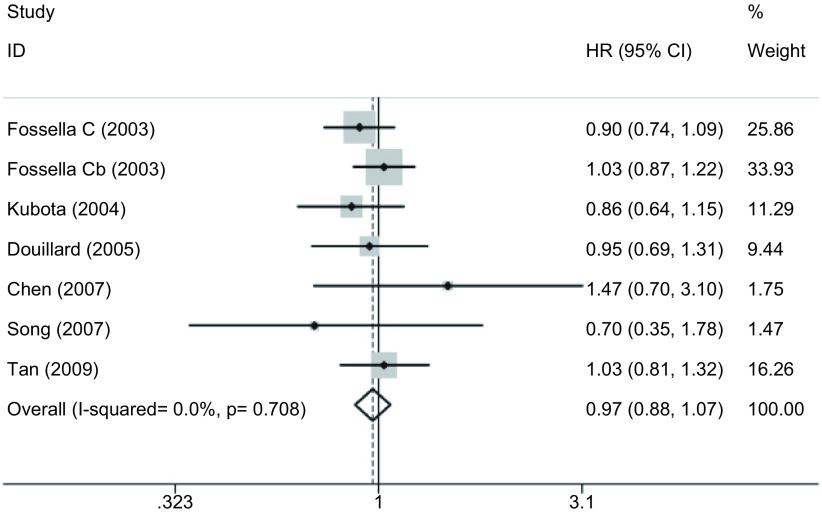
DP方案与VP方案治疗晚期NSCLC的1年生存率的*meta*分析 *Meta*-analysis of one year survive rate for patients with advanced NSCLC treated with DP regimen versus VP regimen

6项研究^[15-20]^报道了1年生存率，各研究间无统计学异质性（*P*=0.708, *I*^2^=0.0%），采用固定效应模型，*meta*分析结果显示DP方案与VP方案在1年生存率方面的差异无统计学意义（HR=0.968, 95%CI: 0.877-1.068, *P*=0.514）。

#### 2.3.2 2年生存率（图 3）

**3:**
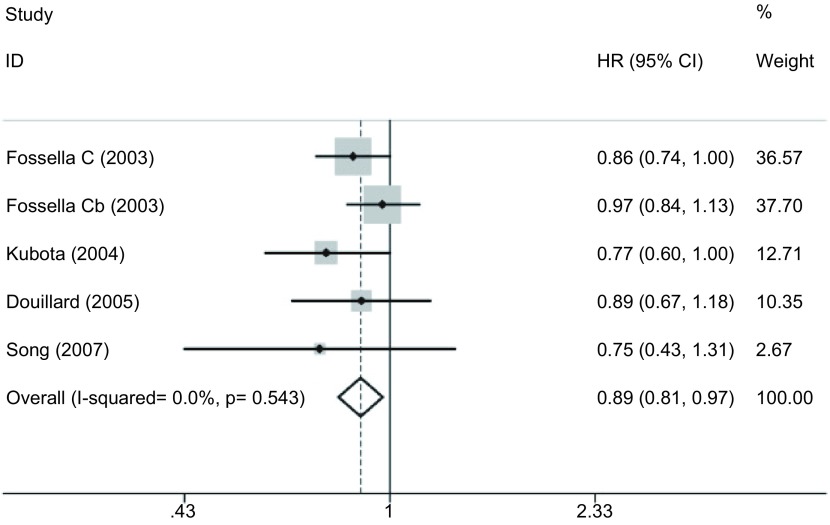
DP方案与VP方案治疗晚期NSCLC的2年生存率的*meta*分析 *Meta*-analysis of two year survive rate for patients with advanced NSCLC treated with DP regimen versus VP regimen

4项研究^[15-17, 19]^报道了2年生存率，各研究间无统计学异质性（*P*=0.543, *I*^2^=0.0%），采用固定效应模型，*meta*分析结果显示DP方案比VP方案的2年生存率高（HR=0.887, 95%CI: 0.810-0.972, *P*=0.010）。

#### 2.3.3 有效率（图 4）

**4:**
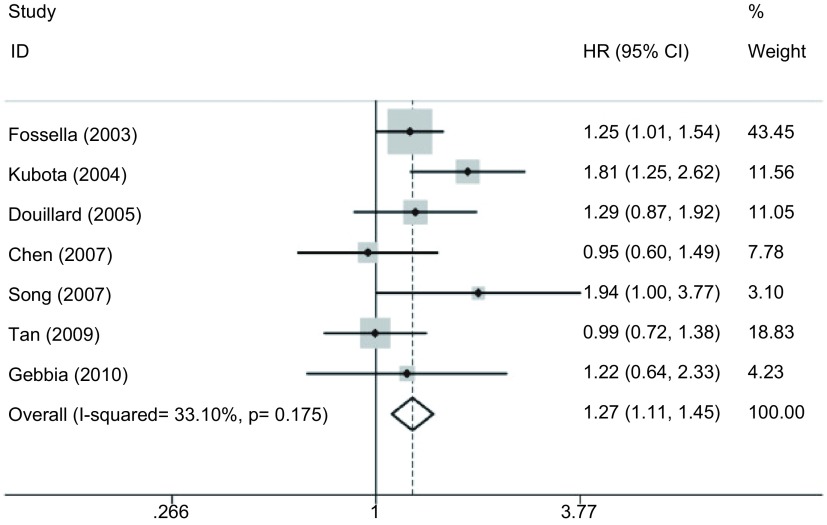
DP方案与VP方案治疗晚期NSCLC的有效率的*meta*分析 *Meta*-analysis of response rate for patients with advanced NSCLC treated with DP regimen versus VP regimen

7项研究^[15-21]^均报道了有效率，各研究间无统计学异质性（*P*=0.175, *I*^2^=33.1%），采用固定效应模型，*meta*分析结果显示DP方案的有效率较VP方案的高（RR=1.276, 95%CI: 1.107-1.450, *P*=0.001）。

#### 2.3.4 毒副作用

##### 2.3.4.1 3级/4级血液学毒性

###### 2.3.4.1.1 3级/4级白细胞减少

6项研究^[15, 16, 18-21]^报道了3级/4级白细胞减少，各研究间存在统计学异质性（*P*=0.065, *I*^2^=51.5%），采用随机效应模型，*meta*分析结果显示DP方案3级/4级白细胞减少发生率与VP方案相比差异无统计学意义（RR=0.861, 95%CI: 0.709-1.047, *P*=0.133）。

###### 2.3.4.1.2 3级/4级中性粒细胞减少

7项研究^[15-21]^均报道了3级/4级中性粒细胞减少，各研究间存在统计学异质性（*P*=0.037, *I*^2^=55.3%），采用随机效应模型，*meta*分析结果显示两方案在3级/4级中性粒细胞减少方面差异无统计学意义（RR=0.926, 95%CI: 0.833-1.029, *P*=0.152）。

###### 2.3.4.1.3 3级/4级血小板减少

7项研究^[15-21]^均报道了3级/4级血小板减少，各研究间无统计学异质性（*P*=0.386, *I*^2^=4.7%），采用固定效应模型，*meta*分析结果显示两方案在3级/4级血小板减少方面差异无统计学意义（RR=0.945, 95%CI: 0.590-1.513, *P*=0.813）。

###### 2.3.4.1.4 3级/4级贫血

7项研究^[15-21]^均报道了3级/4级贫血，各研究间无统计学异质性（*P*=0.275, *I*^2^=20.3%)，采用固定效应模型，*meta*分析结果显示DP方案的3级/4级贫血的发生率低于VP方案，差异有统计学意义（RR=0.386, 95%CI: 0.311-0.478, *P* < 0.001）。

##### 2.3.4.2 3级/4级消化道毒性

###### 2.3.4.2.1 3级/4级厌食

3项研究^[15, 16, 20]^均报道了3级/4级厌食，各研究间存在统计学异质性（*P*=0.074, *I*^2^=61.6%），采用随机效应模型，*meta*分析结果显示两方案在3/4级厌食方面差异无统计学意义（RR=1.293, 95%CI: 0.671-2.492, *P*=0.443）。

###### 2.3.4.2.3 3级/4级恶心

4项研究^[15, 17, 18, 20]^均报道了3级/4级恶心，各研究间存在统计学异质性（*P*=0.001, *I*^2^=82.1%），采用随机效应模型，*meta*分析结果显示两方案在3级/4级恶心方面差异无统计学意义（RR=1.001, 95%CI: 0.392-2.555, *P*=0.998）。

###### 2.3.4.2.4 3级/4级呕吐

6项研究^[15, 17-21]^均报道了3级/4级呕吐，各研究间存在统计学异质性（*P*=0.013, *I*^2^=65.5%），采用随机效应模型，*meta*分析结果显示两方案在3级/4级呕吐方面差异无统计学意义（RR=0.753, 95%CI: 0.386-1.468, *P*=0.404）。

###### 2.3.4.2.5 3级/4级腹泻

6项研究^[15-18, 20, 21]^均报道了3级/4级腹泻，各研究间无统计学异质性（*P*=0.403, *I*^2^=0.5%），采用固定效应模型，*meta*分析结果显示DP方案的3级/4级腹泻发生率明显高于VP方案（RR=3.134, 95%CI: 1.918-5.121, *P*=0.000）。

##### 2.3.4.3 3级/4级衰弱乏力

4项研究^[15, 17, 18, 20]^均报道了3级/4级衰弱乏力，各研究间无统计学异质性（*P*=0.409, *I*^2^=0），采用固定效应模型，*meta*分析结果显示两方案在3级/4级衰弱乏力方面差异无统计学意义（RR=0.899, 95%CI: 0.691-1.170, *P*=0.430）。

### 2.4 发表偏倚分析

在本*meta*分析中，DP方案与VP方案的有效率在所有纳入研究中均有报道，为全面反映纳入研究的情况，最终采用有效率对纳入文献进行漏斗图分析。使用RR值的自然对数及其标准误创建基于*Begg’s*检验为基础的漏斗图来衡量发表偏倚（图 5），该漏斗图呈现出良好的对称性，说明不存在明显的发表偏倚，*Egger’s*检验的结果也证实了漏斗图的结果（*t*=0.44, *P*=0.675）。

**5:**
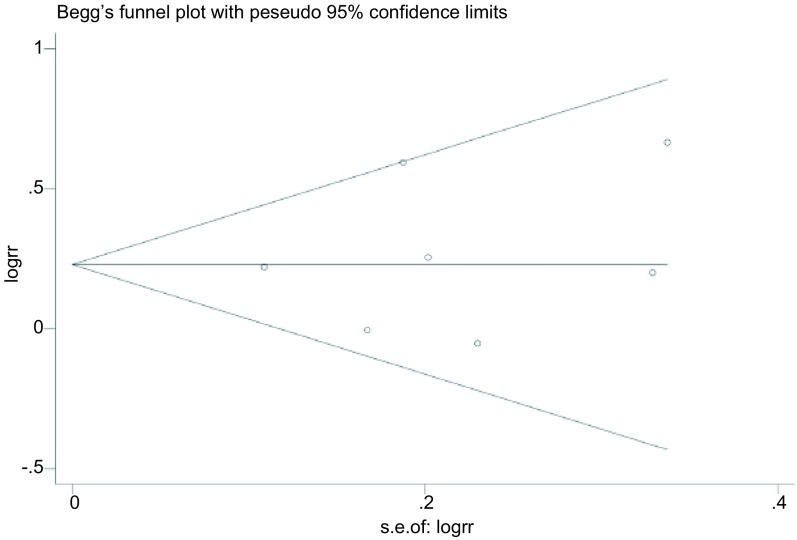
*Begg’s*法检测发表偏倚的漏斗图 The funnel plot of publication bias

## 3 讨论

1995年，NSCLC协作组的*meta*分析^[3]^显示，以铂类为基础药物的化疗比以非铂类药物化疗有效优势比提高62%，1年生存率提高5%，从此确立了以铂类为基础的化疗在治疗NSCLC中的地位。长春瑞滨和多西他赛是第三代新药中具有代表性的两种。

长春瑞滨是一种半合成的长春生物碱类抗肿瘤药物，作用机制为抑制微管蛋白聚合形成微管和诱导微管的解聚，使细胞有丝分裂过程中微管形成障碍而停止在有丝分裂中期，是抗有丝分裂的细胞周期特异性药物^[22]^。药物代谢动力学表明，长春瑞滨是一种广谱的抗癌活性药物，在肺内呈持续高浓度，其单药治疗晚期NSCLC的有效率为12.5%-30%^[23]^。铂类属于细胞非特异性药物，可作用于细胞周期各时像，故与长春瑞滨有协同作用^[24]^。

多西他赛属于半合成的紫杉类新型抗癌药物，其作用机制是通过诱导小管聚合同时抑制已形成的微管束解聚，形成稳定的非功能性微管束，阻断细胞周期的M期和G_2_期，抑制癌细胞的有丝分裂，同为细胞周期特异性药物^[25]^。其与微管蛋白的结合能力是紫杉醇的两倍，而且多西他赛与紫杉醇有不完全交叉耐药，也就是紫杉醇治疗失败的患者，仍可能对多西他赛有效^[26]^。多西他赛同样对多种肿瘤具有抗癌作用，多西他赛单药一线治疗NSCLC的有效率为20%-38%^[27]^。

本*meta*分析结果显示：在疗效方面，DP方案与VP方案在1年生存率方面无统计学差异，但DP方案提高了2年生存率和有效率；在血液学毒副作用方面，两方案在3级/4级白细胞减少、3级/4级中性粒细胞减少、3级/4级血小板减少方面的差异无统计学意义，但DP方案明显降低了3级/4级贫血的发生率；在消化道毒性方面，两方案在3级/4级厌食、3级/4级恶心、3级/4级呕吐方面的差异无统计学意义，但DP方案明显增加了3级/4级腹泻的发生率；两方案在3级/4级衰弱乏力方面的差异无统计学意义。虽然长春瑞滨和多西他赛都是细胞周期特异性药物，但DP方案却提高了2年生存率和有效率且降低了贫血的发生率；虽然DP方案增加了腹泻的发生率，但腹泻在临床较易控制。综合分析DP方案优于VP方案，可作为一线治疗晚期NSCLC的方案推荐临床使用，使广大患者从中获益，但远期疗效有待于高质量、多中心、大样本的RCTs进一步证实。

本*meta*分析的局限性在于：①虽然*Begg’s*检验显示本*meta*分析不存在明显发表偏倚，但只有两项研究^[15, 21]^提及具体随机方法，随机序号均由计算机数字生成器产生。7项研究均未提及分配隐藏。即使使用了正确的随机方法，如果未对随机序列进行合理隐藏，依然会在纳入患者时产生选择偏倚，影响研究结果的真实性。本研究中部分指标，如有效率、厌食、恶心、呕吐、腹泻、衰弱乏力等均为主观测量指标，其真实的测量结果依赖于该指标测量人员是否正确使用盲法，纳入的7项研究中仅有一项研究^[16]^采用双盲，因此可能存在实施和测量偏倚。1项研究^[19]^未报道数据缺失且未进行ITT，可能引起失访偏倚；②7项研究中DP方案与VP方案的化疗剂量和周期不尽相同，可能对最终测量结果有影响；③纳入各研究的基线不完全相同对结果可能存在影响，纳入的文献来自美国、日本、法国、中国、新加坡、意大利，尚需其他国家和地区高质量的RCTs进一步证实；④纳入研究的病例数有限，且部分评价指标存在异质性，如3级/4级白细胞减少、3级/4级中性粒细胞减少、3级/4级厌食、3级/4级恶心及3级/4级呕吐，可能对结果的客观性有一定影响；⑤仅有一项研究^[18]^报道了经济学指标，建议今后临床研究：①试验前进行严格的试验设计，以减少偏倚；②采用客观、国际认可的终点疗效指标，并按照CONSORT标准报告试验结果。

综上所述，DP与VP方案一线治疗晚期NSCLC时，DP方案不但提高了2年生存率和有效率，而且降低了3级/4级贫血的发生率，因此本*meta*分析的结果倾向于DP方案优于VP方案。本*meta*分析的结果或许可为治疗晚期NSCLC提供参考依据。

## References

[1] Jemal A, Bray F, Center MM (2011). Global cancer statistics. CA Cancer J Clin.

[2] Novello S, Le Chevalier T (2003). Chemotherapy for non-small-cell lung cancer. Part 1: Early-stage disease. Oncology (Williston Park).

[3] Alberti W (1995). Chemotherapy in non-small cell lung cancer: a *meta*-analysis using updated data on individual patients from 52 randomised clinical trials. Non-small Cell Lung Cancer Collaborative Group. BMJ.

[4] Hotta K, Matsuo K, Ueoka H (2004). Addition of platinum compounds to a new agent in patients with advanced non-small-cell lung cancer: a literature based *meta*-analysis of randomised trials. Ann Oncol.

[5] Le Chevalier T, Brisgand D, Douillard JY (1994). Randomized study of vinorelbine and cisplatin versus vindesine and cisplatin versus vinorelbine alone in advanced non-small-cell lung cancer: results of a European multicenter trial including 612 patients. J Clin Oncol.

[6] Wozniak AJ, Crowley JJ, Balcerzak S P (1998). Randomized trial comparing cisplatin with cisplatin plus vinorelbine in the treatment of advanced non-small-cell lung cancer: a Southwest Oncology Group study. J Clin Oncol.

[7] Kelly K, Crowley J, Bunn P J (2001). Randomized phase Ⅲ trial of paclitaxel plus carboplatin versus vinorelbine plus cisplatin in the treatment of patients with advanced non-small-cell lung cancer: a Southwest Oncology Group trial. J Clin Oncol.

[8] Le Chevalier T, Monnier A, Douillard JY (1998). Docetaxel (Taxotere) plus cisplatin: an active and well-tolerated combination in patients with advanced non-small cell lung cancer. Eur J Cancer.

[9] Zalcberg J, Millward M, Bishop J (1998). Phase Ⅱ study of docetaxel and cisplatin in advanced non-small-cell lung cancer. J Clin Oncol.

[10] Belani C, Lynch T (2001). Docetaxel (Taxotere) in combination with platinums in patients with non-small cell lung cancer: trial data and implications for clinical management. Semin Oncol.

[11] Schiller JH, Harrington D, Belani CP (2002). Comparison of four chemotherapy regimens for advanced non-small-cell lung cancer. N Engl J Med.

[12] Therasse P, Arbuck SG, Eisenhauer EA (2000). New guidelines to evaluate the response to treatment in solid tumors. European Organization for Research and Treatment of Cancer, National Cancer Institute of the United States, National Cancer Institute of Canada. J Natl Cancer Inst.

[b13] 13Higgins JPT, Green S. Cochrane Handbook For Systematic Reviews of Interventions Version 5. 1. 0 [Updated March 2011]. The Cochrane Collaboration, 2011. Available from: www. cochrane-handbook. org.

[14] Egger M, Davey SG, Schneider M (1997). Bias in *meta*-analysis detected by a simple, graphical test. BMJ.

[15] Fossella F, Pereira JR, von Pawel J (2003). Randomized, multinational, phase Ⅲ study of docetaxel plus platinum combinations versus vinorelbine plus cisplatin for advanced non-small-cell lung cancer: the TAX 326 study group. J Clin Oncol.

[16] Kubota K, Watanabe K, Kunitoh H (2004). Phase Ⅲ randomized trial of docetaxel plus cisplatin versus vindesine plus cisplatin in patients with stage Ⅳ non-small-cell lung cancer: the Japanese Taxotere Lung Cancer Study Group. J Clin Oncol.

[17] Douillard JY, Gervais R, Dabouis G (2005). Sequential two-line strategy for stage Ⅳ non-small-cell lung cancer: docetaxel-cisplatin versus vinorelbine-cisplatin followed by cross-over to single-agent docetaxel or vinorelbine at progression: final results of a randomised phase Ⅱ study. Ann Oncol.

[18] Chen YM, Perng RP, Shih J F (2007). A randomized phase Ⅱ study of docetaxel or vinorelbine in combination with cisplatin against inoperable, chemo-naive non-small-cell lung cancer in Taiwan. Lung Cancer.

[19] Song HP, Qiu WS, Xu JH (2007). First-line chemotherapy of dcoetaxel/cisplatin for advanced non-small cell lung cancer. Zhongguo Zhong Liu Lin Chuang.

[20] Tan EH, Rolski J, Grodzki T (2009). Global Lung Oncology Branch trial 3 (GLOB3): final results of a randomised multinational phase Ⅲ study alternating oral and *i.v.* vinorelbine plus cisplatin versus docetaxel plus cisplatin as first-line treatment of advanced non-small-cell lung cancer. Ann Oncol.

[21] Gebbia V, Lorusso V, Galetta D (2010). First-line cisplatin with docetaxel or vinorelbine in patients with advanced non-small-cell lung cancer: a quality of life directed phase Ⅱ randomized trial of Gruppo Oncologico Italia Meridionale. Lung Cancer.

[22] Lobert S, Vulevic B, Correia JJ (1996). Interaction of vinca alkaloids with tubulin: a comparison of vinblastine, vincristine, and vinorelbine. Biochemistry.

[23] Le Chevalier T, Brisgand D, Douillard JY (1994). Randomized study of vinorelbine and cisplatin versus vindesine and cisplatin versus vinorelbine alone in advanced non-small-cell lung cancer: results of a European multicenter trial including 612 patients. J Clin Oncol.

[24] Baron MG, Feliu J, Ciron GG (1995). Phase Ⅱ trial of mitomycin, vinorelbine and cisplatin in non-small-cell lung cancer (NSCLC). Proc Am Soc Clin Oncol.

[25] Horwitz SB, Cohen D, Rao S (1993). Taxol: mechanisms of action and resistance. J Natl Cancer Inst Monogr.

[26] Davies AM, Lara PJ, Mack PC (2003). Docetaxel in non-small cell lung cancer: a review. Expert Opin Pharmacother.

[27] Comer AM, Goa KL (2000). Docetaxel: a review of its use in non-small cell lung cancer. Drugs Aging.

